# Supplementary figures and images for: AtPIG-S, a predicted Glycosylphosphatidylinositol Transamidase subunit, is critical for pollen tube growth in Arabidopsis
Source: BMC Plant Biol. 2020 Aug 18;20:380. doi: 10.1186/s12870-020-02587-x (PMC7437025; doi:10.1186/s12870-020-02587-x)

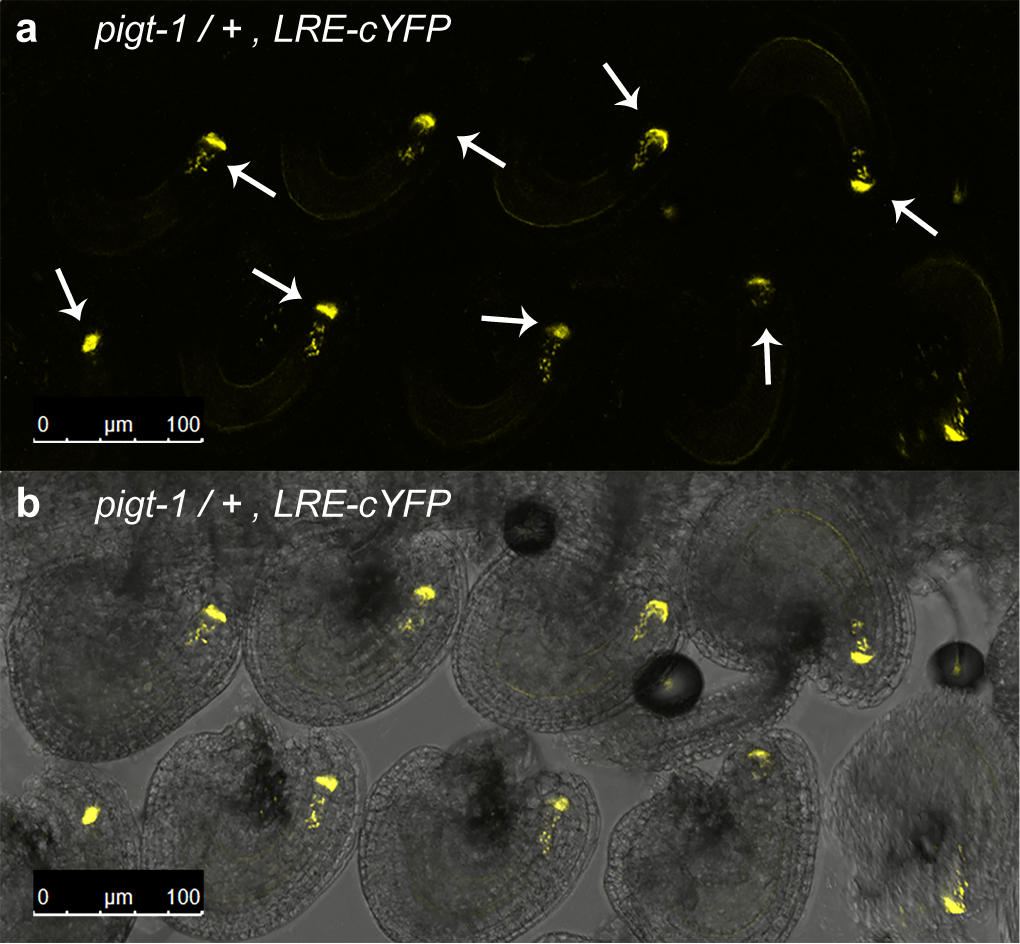

Supplement: Supplementary file 1 — Additional file 1: pigt-1 mutation does not affect polar localization of LRE-cYFP in the filiform apparatus of synergid cells. [file 12870_2020_2587_MOESM1_ESM.tif]

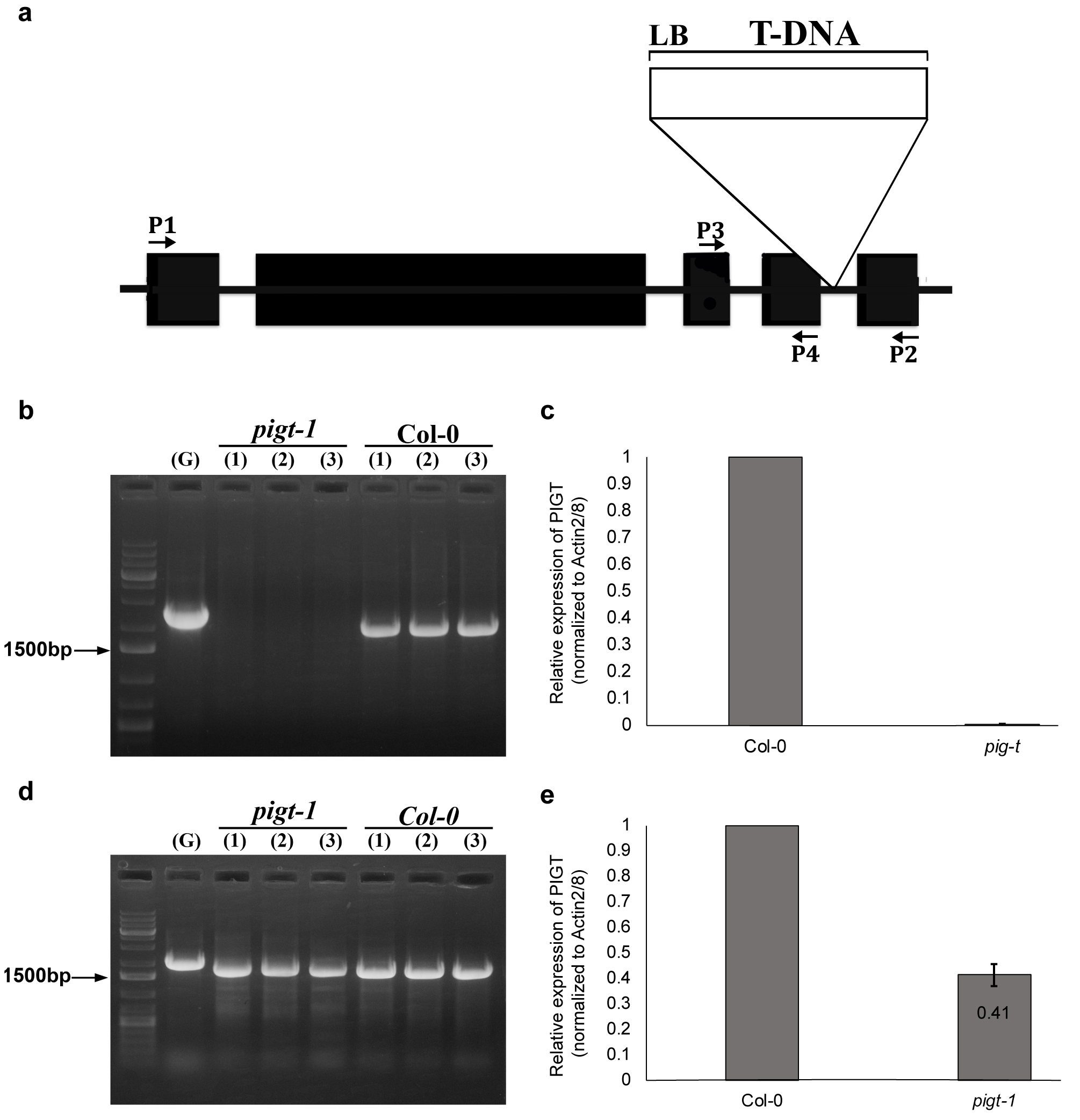

Supplement: Supplementary file 2 — Additional file 2: Analysis of PIG-T expression in the pigt-1 mutant. [file 12870_2020_2587_MOESM2_ESM.tif]

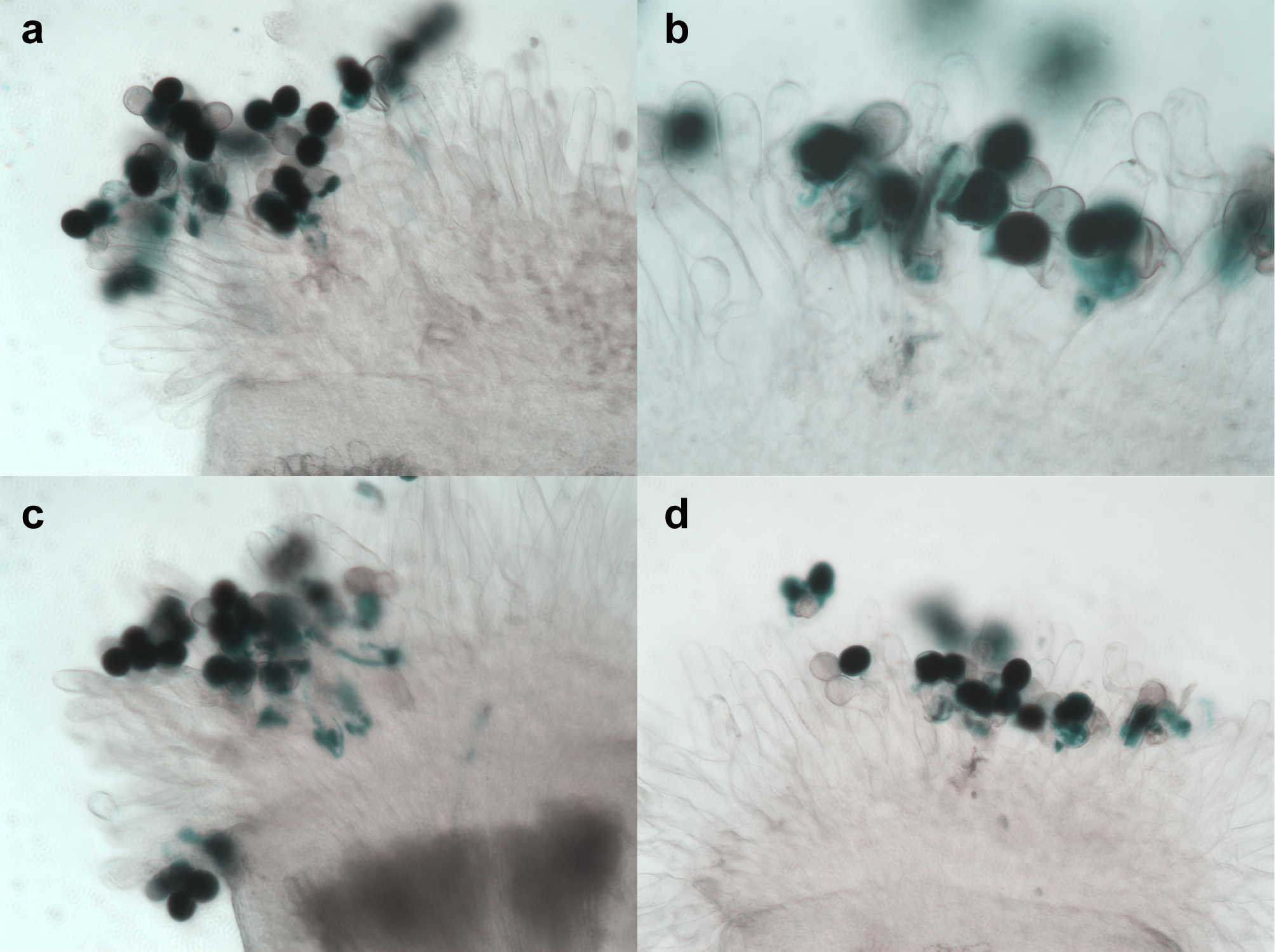

Supplement: Supplementary file 3 — Additional file 3: Pollen germination and tube emergence defects. [file 12870_2020_2587_MOESM3_ESM.tif]

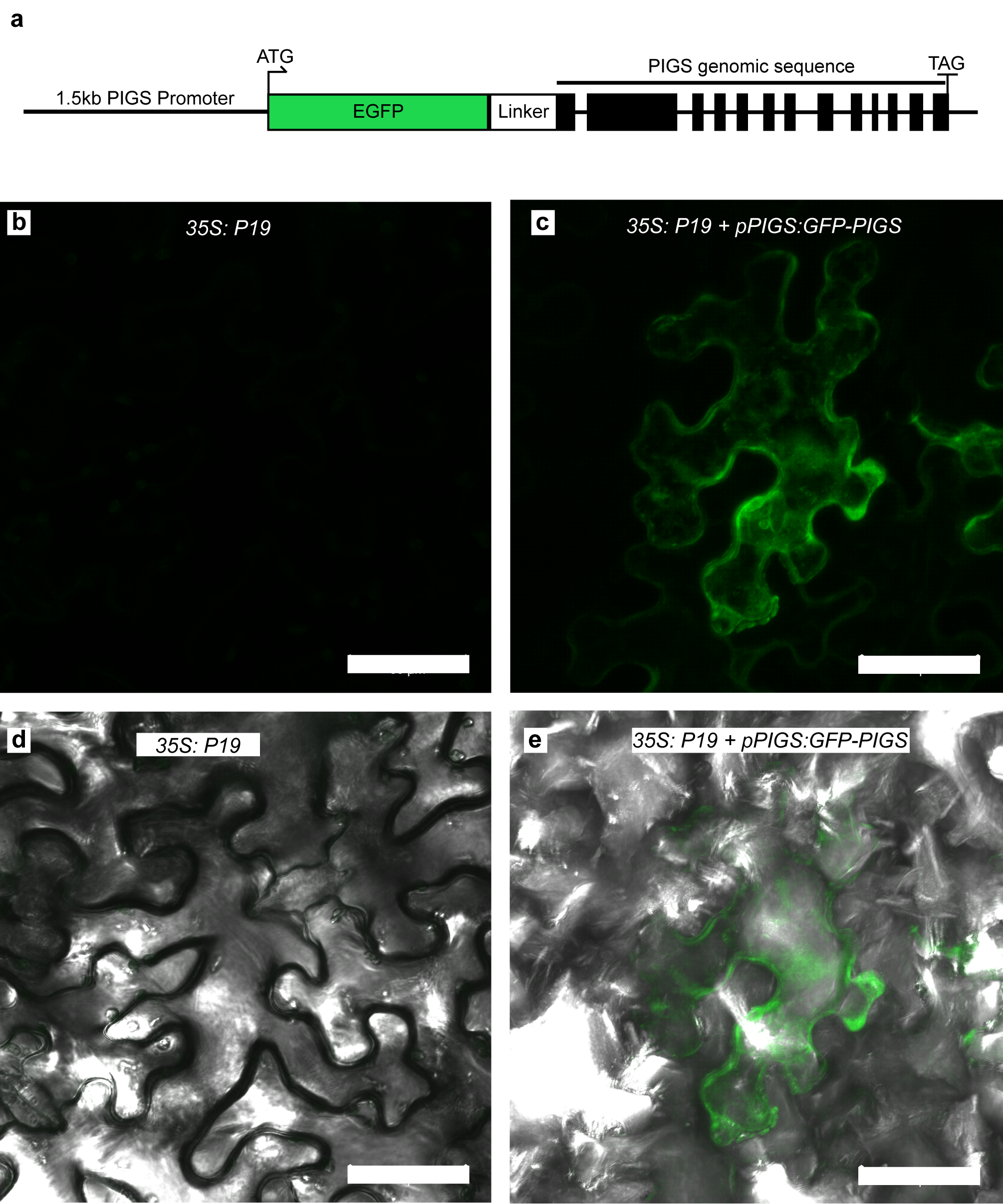

Supplement: Supplementary file 4 — Additional file 4: Transient expression of AtGFP-PIGSprotein in Nicotiana benthamiana leaves. [file 12870_2020_2587_MOESM4_ESM.tif]

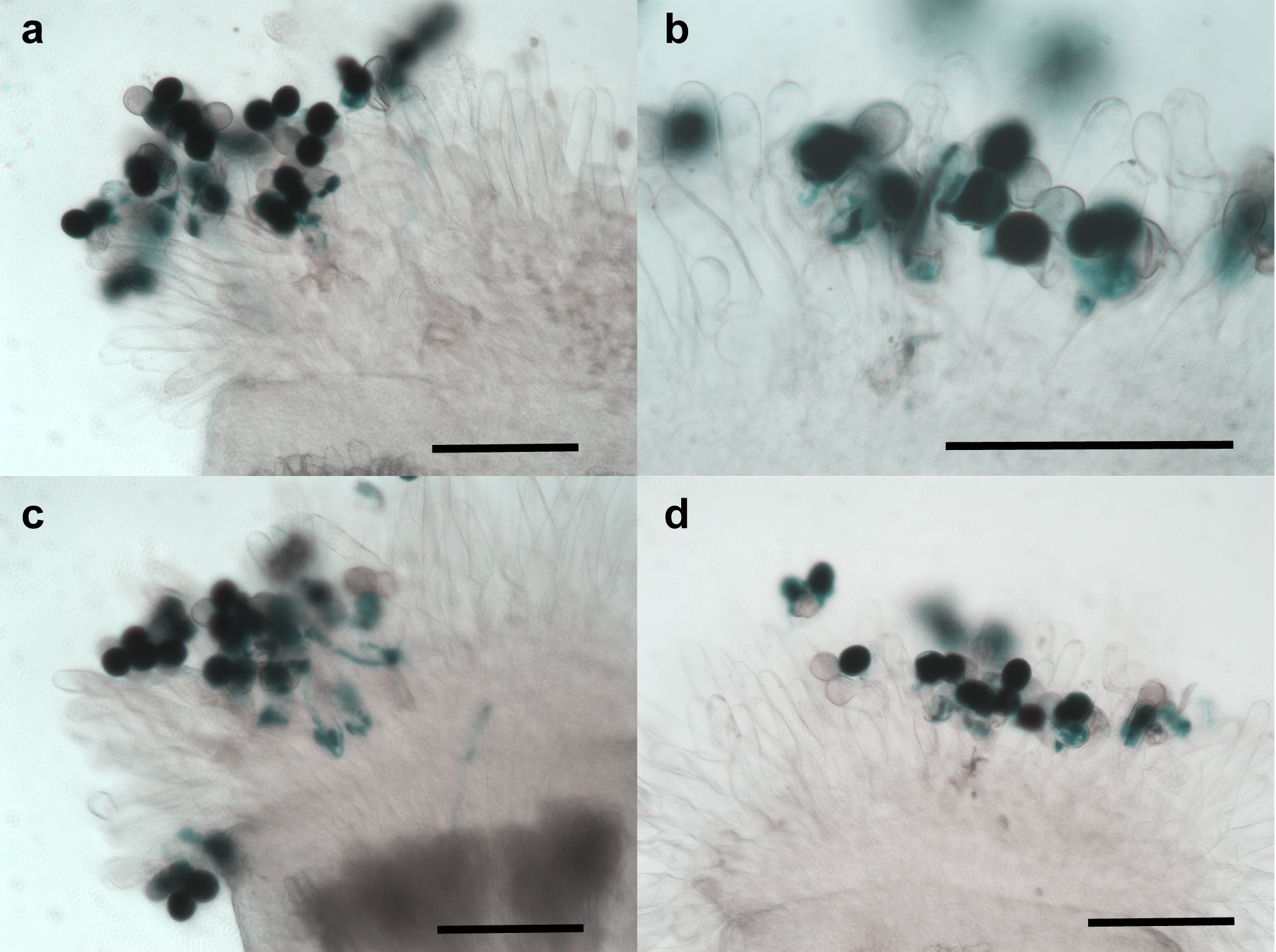

Supplement: Supplementary file 5 — Additional file 5: Selfed seed set in T2 pigs-1/+, pPIGS:GFP-PIGS plants. [file 12870_2020_2587_MOESM5_ESM.tif]

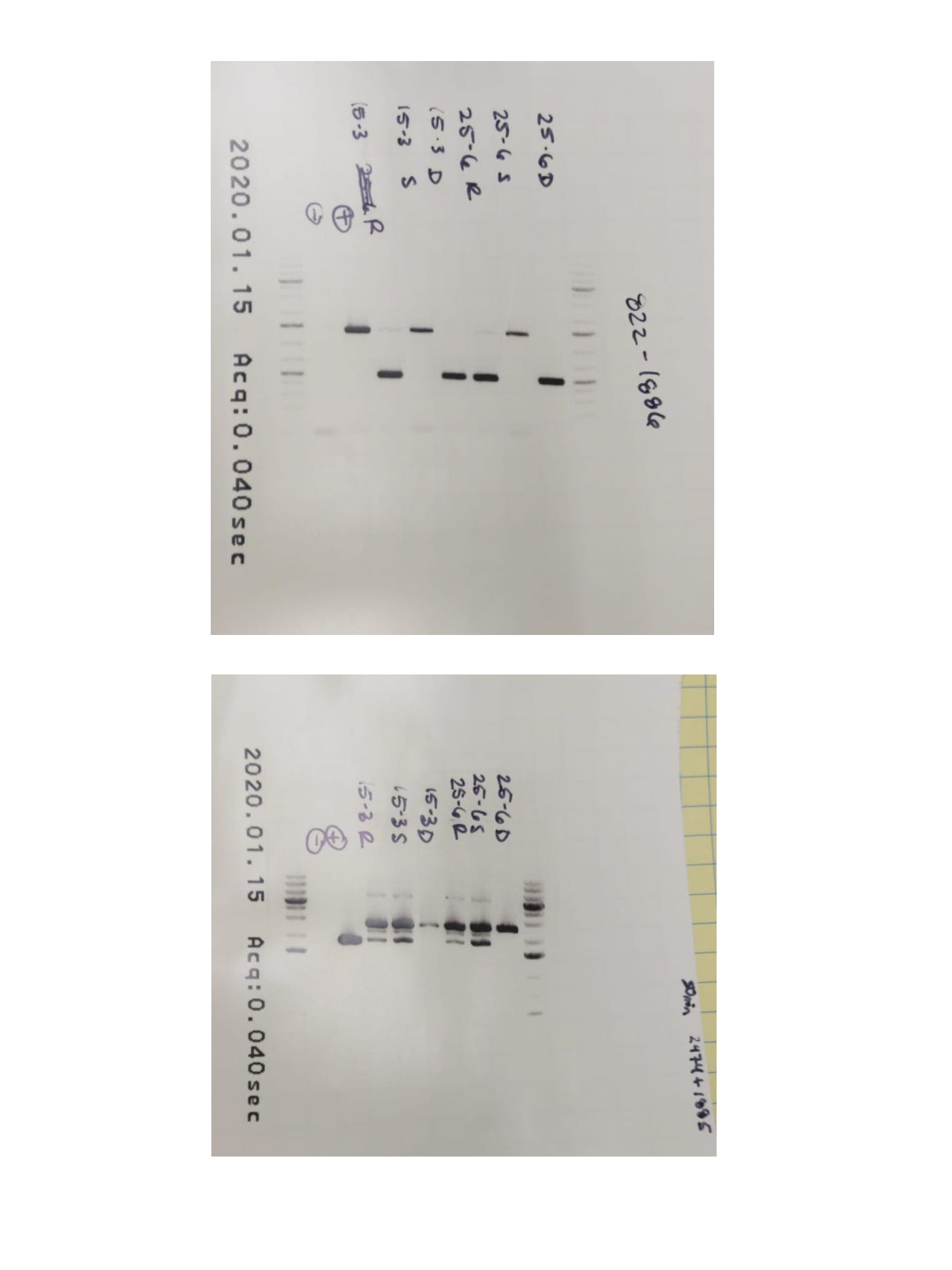

Supplement: Supplementary file 6 — Additional file 6: PCR-based genotyping of pigs-1/pigs-1, pPIGS:GFP-PIGS plants. [file 12870_2020_2587_MOESM6_ESM.tiff]
