# Supplementary material for: AtPIG-S, a predicted Glycosylphosphatidylinositol Transamidase subunit, is critical for pollen tube growth in Arabidopsis
Source: BMC Plant Biol. 2020 Aug 18;20:380. doi: 10.1186/s12870-020-02587-x (PMC7437025; doi:10.1186/s12870-020-02587-x)
Supplement: Supplementary file 7 — Additional file 7: Table S1. List of primers used in this study. [file 12870_2020_2587_MOESM7_ESM.docx]

| **Additional Table 1. List of primers used in this study** | | | | | |
| --- | --- | --- | --- | --- | --- |
| Primer  # | Primer  Name | Sequence (5' - 3') | Template | Primer  Pair | Expected Length  (bp) |
| *pPIGS::GFP-PIGS* construct | | | | |  |
| 2366 | PIGS Promoter_F | CCCATATGGTCGACCTGCCTCATGAATATCATTAAGATCCAGA | Col-0 genomic DNA | 2366 + 2368 | 1534 |
| 2368 | PIGS Promoter_R | CGCCCTTGCTCACCATCGGTGCTTTCCGAGAG |  |  |  |
| 2367 | GFP_F | CTCTCGGAAAGCACCGATGGTGAGCAAGGGCG | pCAMBIA1300-GFP | 2367 + 2369 | 756 |
| 2369 | GFP_R | CCACCTCCACCTCCAGGCCGGCCCTTGTACAGCTCGTCCATGCCG |  |  |  |
| 2370 | PIGS_F | GCCTGGAGGTGGAGGTGGAGCTATGGAAGAAATCTCCGATCGT | Col-0 genomic DNA | 2370 + 2371 | 3280 |
| 2371 | PIGS 3’UTR_R | GTCGGCGCGCCCACCGGTTAGACATCTGTAATCGTAAAC |  |  |  |
| *PIGT* RT-PCR | | | | |  |
| 2364 | PIGT Full Length Forward (P1) | ATGGCTAGTCTTCTTCGATCC | Col-0 genomic DNA  cDNA | 2364 + 2365 | Genomic: 2355  cDNA: 1935 |
| 2365 | PIGT Full Length Reverse (P2) | CTACTCGTCCGTGGAAAAATATTG |  |  |  |
| 2364 | PIGT Full Length Forward (P1) | ATGGCTAGTCTTCTTCGATCC | Col-0 genomic DNA  cDNA | 2364 + 2365 | Genomic: 2063  cDNA: 1747 |
| 2381 | PigT_exon4R (P4) | CTTGGCTTTTGAGAAACCTTTC |  |  |  |
| 464 | ACTIN2/8_F | CCTATTGAGCATGGTGTTGTTAGCAAC | Col-0 genomic DNA  cDNA | 464 + 465 | Genomic: 373  cDNA:277 |
| 465 | ACTIN2/8_R | TGTGAGACACACCATCACCAGA |  |  |  |
| *PIGT* qRT-PCR | |  |  |  |  |
| 1890 | AT3G07140-1702F (P3) | CTTTGATAAGCTTCCCCGATC | cDNA | 1890 + 2365 | 452 |
| 2365 | PIGT Full Length Reverse (P2) | CTACTCGTCCGTGGAAAAATATTG |  |  |  |
| 1890 | AT3G07140-1702F (P3) | CTTTGATAAGCTTCCCCGATC | cDNA | 1890 + 2381 | 264 |
| 2381 | PigT_exon4R (P4) | CTTGGCTTTTGAGAAACCTTTC |  |  |  |
| 464 | ACTIN2/8_F | CCTATTGAGCATGGTGTTGTTAGCAAC | cDNA | 464 + 465 | 277 |
| 465 | ACTIN2/8_R | TGTGAGACACACCATCACCAGA |  |  |  |
| *pigt-1* genotyping | |  |  |  |  |
| 1889 | AT3G07140-2720R  (SALK_099158 LP) | GGATGCAACAAGAGAAAGCTG | *pigt-1* genomic DNA | 1889 + 1890 | 1019 |
| 1890 | AT3G07140-1702F  (SALK_099158 RP) | CTTTGATAAGCTTCCCCGATC |  |  |  |
| 383 | SALK-LB-6108R  (pBIN-pROK2 LB) | CCAGCCAACAGCTCCCCGAC | *pigt-1* genomic DNA | 383 + 1890 | ~600 |
| 1890 | AT3G07140-1702F  (SALK_099158 RP) | CTTTGATAAGCTTCCCCGATC |  |  |  |
| *pigs-1* and *PIGS* genotyping | |  |  |  |  |
| 1885 | AT3G07180-1700R  (SAIL_162_D06_LP) | GAAGGACATATTGACGCAAGG | *pigs-1* genomic DNA | 1885 + 1886 | 1042 |
| 1886 | AT3G07180-658F  (SAIL_162_D06_RP) | TAAAGAGAATGCCAATGGTGG |  |  |  |
| 822 | SAIL-LB-451R  (pDAP101 LB) | GCCTTTTCAGAAATGGATAAATAGCCTTGCTTCC | *pigs-1* genomic DNA | 822 + 1886 | ~500 |
| 1886 | AT3G07180-658F  (SAIL_162_D06_RP) | TAAAGAGAATGCCAATGGTGG |  |  |  |
| 1885 | AT3G07180-1700R  (SAIL_162_D06_LP) | GAAGGACATATTGACGCAAGG | *pigs-1* genomic DNA | 1885 + 2474 | PIGS-GFP: 2586  PIGS: 1896 |
| 2474 | PIGS Promoter | CGCTGAGCTAAGACGGCTATTG |  |  |  |
